# Mechanical and Material Analysis of 3D-Printed Temporary Materials for Implant Reconstructions—A Pilot Study

**DOI:** 10.3390/biomedicines12040870

**Published:** 2024-04-15

**Authors:** Adam Nowicki, Karolina Osypko, Adam Kurzawa, Maciej Roszak, Karina Krawiec, Dariusz Pyka

**Affiliations:** 1 Diamante Dental Clinic, ul. Sportowa 48A/C, 59-300 Lubin, Poland; adamusnowicki@gmail.com; 2 Dental Salon, Oral Surgery Academy, ul. E. Horbaczewskiego 53A, 54-130 Wroclaw, Poland; 3 Department of Lightweight Elements Engineering, Foundry and Automation, Faculty of Mechanical Engineering, Wroclaw University of Science and Technology, Smoluchowskiego 25, 50-370 Wroclaw, Poland; adam.kurzawa@pwr.edu.pl; 4 Department of Mechanics, Materials Science and Biomedical Engineering, Wrocław University of Science and Technology, Smoluchowskiego 25, 50-370 Wroclaw, Poland; maciej.roszak@pwr.edu.pl (M.R.); 254762@student.pwr.edu.pl (K.K.); dariusz.pyka@pwr.edu.pl (D.P.)

**Keywords:** denture, 3D printing, temporary prosthesis, resin, mechanical properties, numerical analysis

## Abstract

In this study, the authors analyzed modern resin materials typically used for temporary reconstructions on implants and manufactured via 3D printing. Three broadly used resins: NextDent Denture 3D, NextDent C&B MFH Bleach, and Graphy TC-80DP were selected for analysis and compared to currently used acrylic materials and ABS-like resin. In order to achieve this, mechanical tests were conducted, starting with the static tensile test PN-EN. After the mechanical tests, analysis of the chemical composition was performed and images of the SEM microstructure were taken. Moreover, numerical simulations were conducted to create numerical models of materials and compare the accuracy with the tensile test. The parameters obtained in the computational environment enabled more than 98% correspondence between numerical and experimental charts, which constitutes an important step towards the further development of numeric methods in dentistry and prosthodontics.

## 1. Introduction

Nowadays, the challenges of reconstructing and treating stomatognathic conditions are becoming more demanding; however, at the same time, new opportunities are opening up thanks to advances in technology and innovations in biomedical materials [1,2,3]. Implantology, as a branch of medicine dealing with the placement of implants and occlusal reconstruction, is growing in importance, offering increasingly advanced solutions for patients with various types of injuries, diseases, or bone tissue defects. Another rapidly growing field of dentistry is prosthodontics, which provides various solutions for people with missing teeth using different types of prostheses such as fixed or removable dentures, crowns, or bridges [4,5]. With advances in technology and research, the validity of collaboration between fields such as prosthodontics and engineering in developing innovative solutions that can revolutionize the field of dentistry is being emphasized. Collaboration between prosthodontists and engineers enables the use of advanced technologies, such as 3D printing and laser scanning, to accurately design and create dentures.

Three-dimensional printing technology provides the unique ability to quickly and precisely create structures with complex geometries, making it an ideal tool for creating custom prosthetic solutions, in this case dentures, tailored to individual patients’ needs. Incremental technologies are additive manufacturing methods, wherein the product is produced layer by layer, thus allowing for a greater range of personalization possibilities both in terms of the material used, the type of product, and its aesthetic qualities. However, to achieve the full potential of this technology, it is necessary to use appropriate biomedical materials, e.g., thermoplastic polymers, ceramics, or metals with properties that meet stringent clinical requirements for, e.g., biocompatibility [6].

In this context, research into the mechanical and material properties of biomedical materials is crucial for ensuring the safety, effectiveness, and durability of implant restorations used in clinical practice. With such an aim, this article is devoted to a detailed analysis of three selected biomedical materials that are used in implantology and prosthodontics: NextDent Denture 3D, NextDent C&B MFH Bleach, and Graphy TC-80DP. Acrylic dentures are very popular among types of removable prosthetic restorations, hence acrylic is our reference material. They are characterized by a complete structure usually made of a single material. They efficiently mimic both teeth and soft tissues, so that the entire prosthesis successfully restores patients’ aesthetics and chewing function [7].

There are many studies in the literature confirming the biocompatibility of resins adapted to 3D printers for use in prosthetics, but there are no available studies focusing on their exact mechanical properties that take into account the conditions of their working environment [8,9,10]. A group of German researchers conducted a study of base resins for dental prostheses fabricated by 3D printing, milling, and conventional polymerization to examine water sorption, modulus of elasticity, and flexural strength. One of the tested materials was NextDent Denture 3D resin. The results showed that samples produced by 3D printing with the base resin exhibited higher water sorption and water solubility, but also higher elastic modulus and flexural strength values than the control groups. A study by a Chinese group on the comparison of flexural properties and cytotoxicity of temporary materials printed on LCD and DLP 3D printers was published in *The Journal Of Prosthetic Dentistry* in 2021 [11]. One of the materials considered was NextDent C&B MFH resin. The specimens were made with a Phrozen Sonic printer and then postpolymerized with a PhrozenCure device (Phrozen Tech. Co., Ltd., Taiwan). After such treatment, the flexural strength of the materials exceeded 50 MPa, which is the minimum requirement for flexural strength in ISO10477 [12]. A group of dentists and engineers in Korea conducted a study on the fracture toughness, biaxial strength (BFS) and dynamic mechanical analysis (DMA) of 3D printing resins for aesthetic restoration of deciduous teeth. The study compared two 3D printing resins, Graphy TC-80DP and NextDent C&B MFH. The biaxial strength of Graphy was higher for all thicknesses than that of NextDent. Both materials showed high survivability (over 90%) at 50 and 150 MPa loads. The study suggests that 3D-printed resins may be an acceptable option for manufacturing permanent dentures of deciduous teeth, distinguished by sufficient strength to withstand chewing forces in children. In addition, these resins can be an alternative to zirconia crowns, providing satisfactory aesthetics and durability.

This article contributes to the discussion on the use of modern technologies in implantology, prosthetics, or restorative surgery, emphasizing the need to constantly seek innovative solutions that can improve the quality of life of patients and the effectiveness of treatment of osteoarticular disorders. Through cooperation between the fields of science, engineering, and clinical practice, we aim to create new standards in the field of implantology that are based on a solid scientific and technological foundation.

## 2. Description of the Problem

The aim of our research is to analyze the mechanical and material properties of a chosen group of materials in order to compare them from the perspective of effectiveness in the production of fixed dentures or temporary prosthesis in implants. The static tensile tests of 3D-printed tensile specimens in dogbone shapes (NextDent Denture 3D, NextDent C&B MFH Bleach, Graphy TC-80DP) were particularly examined to enable validation of numerical models and adjusted rheological models of these materials. Moreover, the fracture surface topography and chemical composition of the resins were examined, and numerical analyses describing quasi-static tensile tests were conducted. This analysis allowed for the selection of a material with the best mechanical properties, which is suitable for requirements set for dentures, which in implantology are usually used as a temporary solution for the osseointegration period.

## 3. Materials and Methods

### 3.1. Materials

**Figure 1:**
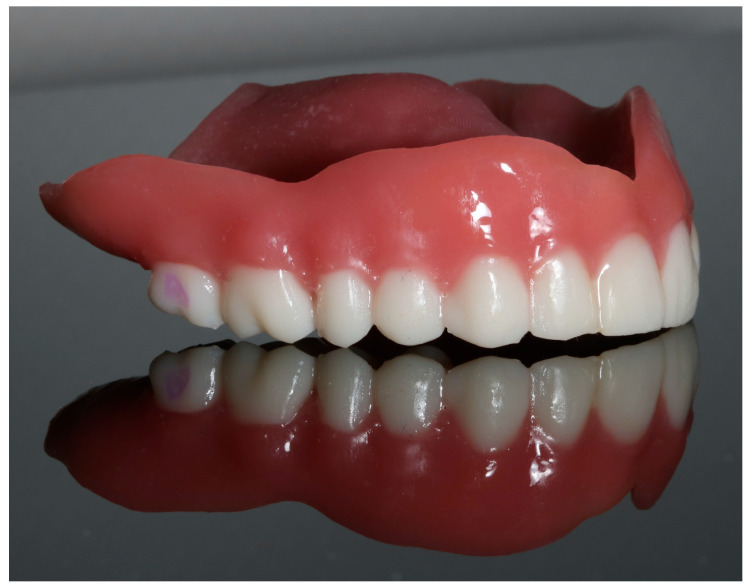
Dentures made with NextDent Denture 3D and NextDent C&B MFH Bleach materials (photography by A.N.).

This research focuses on the analysis of a group of biocompatible photopolymer materials in the form of resins dedicated to additive technologies. Two broadly used materials from the manufacturer NextDent (NextDent B.V., Soesterberg, The Nederlands), Denture 3D and C&B MFH Bleach, and one from Graphy TC-80DP (Graphy Inc., Seoul, Republic of Korea) were selected. Acrylic resin, which is still widely used today as a material for manufacturing removable dentures, was used as the reference material [13]. Figure 1 shows an example of a removable prosthesis made from the resins mentioned above.

NextDent Denture 3D is a Class IIa material, which means it is safe for long-term use in contact within the biological environment and does not cause immune reactions or toxic effects. It is a material used in 3D printing technology for the production of dental prostheses, especially denture plates in the form of bases for a wide range of removable prosthetic restorations. It is available in five color variants corresponding to the natural color of the gums. Thanks to the appealing aesthetics of the manufactured products, they camouflage properly in the oral cavity. Selected values of the material’s mechanical properties given by the manufacturer are shown in Table 1. According to the material’s composition statement, the contents of the individual components are as follows: ethoxylated bisphenol A dimethacrylate accounts for at least 75%; 7,7,9 (or 7,9,9-trimethyl-4,13-dioxo-3,14-dioxo-5, 12-diazahexadecane-1,16-diyl) bismethacrylate is in the range of 10% to 20%; 2-hydroxyethyl methacrylate accounts for 5% to 10%; silicon dioxide and diphenyl (2,4,6-trimethylbenzoyl) phosphine oxide are within a range of 5% to 10% and 1% to 5%, respectively; and titanium dioxide is present in an amount of less than 0.1% [14,15].

Another material from this manufacturer that we chose to analyze is NextDent C&B MFH (Micro Filled Hybrid) Bleach (NextDent B.V., Soesterberg, The Nederlands). This material, thanks to its light color that resembles the natural color of teeth after whitening, ensures a precise fit and strong aesthetic appearance. Using an additive method in the form of 3D printing, it can be successfully implemented to repair or modify existing dentures or to create various prosthetic components such as crowns, bridges, partial dentures, or inlays. NextDent MFH Bleach is a comprehensive dental material that features easy processing and polishing. It can be colored using a variety of composite staining kits to match individual patients’ aesthetic preferences. There is a significant gap in the literature regarding the mechanical properties of this material (Table 2). The manufacturer lists the following compounds in the chemical composition of the resin: 7,7,9 (or 7,9,9)-trimethyl-4, 13-dioxo-3, 14-dioxa-5, 12-diazahexadecane-1, 16-diyl bismethacrylate, ethylene dimethacrylate, 2-hydroxyethyl methacrylate, and diphenyl (2,4,6-trimethylbenzoyl)-phosphine oxide [15,16].

**Figure 2:**
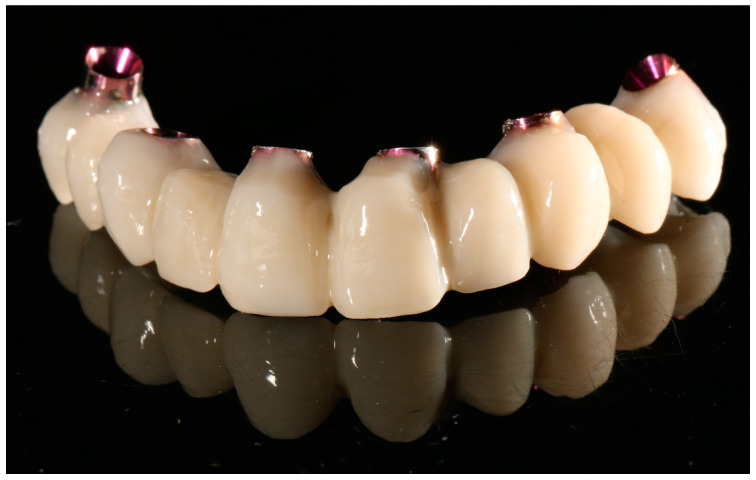
Dental crowns made of Graphy TC-80DP material (photography by A.N.).

Prosthetic components such as crowns and bridges are also made from Graphy TC-80DP resin (Graphy Inc., Seoul, Republic of Korea). Products manufactured with it using 3D printing technology are characterized by high durability and permanent biocompatibility. Due to its good strength and the ease of achieving precision, the material works well for both temporary and permanent restorations [17]. Its selected mechanical properties are shown in Table 3. Its ivory color successfully mimics that of natural teeth. Figure 2 shows a finished temporary bridge made from Graphy TC-80DP (Graphy Inc., Seoul, Republic of Korea). Despite the unavailability of information from the manufacturer about the exact chemical composition of the material, studies show that it is a polyurethane resin-based methacrylate ligomer with phosphine oxides and an additional pigment to provide the ivory color [18].

For nearly 100 years, acrylic and later acrylic resins, whose main component is polymethylmethacrylate (PMMA), have been frequently used as materials for dental prostheses. They are characterized by ease of processing with good mechanical properties and a relatively low price compared to some other dental materials [19]. Based on their construction and dental application, acrylic polymers are divided into hard (brittle) polymers, from which denture bases are manufactured, and soft (flexible) polymers that are used as the lining of denture bases. CAD/CAM acrylic dentures are created from prefabricated dental acrylic blocks and are characterized by optimal mechanical and physical properties. Despite a number of advantages of acrylic polymers, such as adequate hardness, abrasion resistance, or low water absorption, they have disadvantages in the form of limited bending strength; more importantly, the literature mentions allergic reactions in patients and dental staff to potentially toxic substances released during their use [20,21]. Table 4 shows selected mechanical properties of commonly used acrylic resin. 

Compared to traditional acrylic, the aforementioned materials can offer better quality, precision fit, durability, and aesthetics for dental prostheses. However, choosing the right material depends on a number of factors, such as dentist’s and patient’s preferences, type of denture, cost, and availability of technology.

### 3.2. 3D Print Technology

In the field of dentistry and industry, a variety of 3D printing techniques have gained popularity, including stereolithography (SLA), selective laser sintering (SLS) and digital light processing (DLP), among others [22,23,24,25]. The selected photopolymer materials, NextDent Denture 3D, NextDent C&B MFH Bleach (NextDent B.V., Soesterberg, The Nederlands), and Graphy TC-80DP (Graphy Inc., Seoul, Republic of Korea), are used with DLP technology. This method uses UV light emitted by a DLP matrix to cure the liquid resin. The designed layer pattern is projected onto the flat surface of the resin, and the UV light cures the selected areas to form the object layer. This process is repeated until the printing is complete [26].

To make the dogbone shapes for the tensile strength test, two DLP (digital light processing)-based 3D printers were used: Phrozen Mini 8k and Phrozen Sonic Mighty 8k (Phrozen Tech. Co., Ltd., Taiwan). Their use allows high-quality and precision printing to be achieved, which makes them a suitable tool for the production of diagnostic models as well as finished products such as dental prostheses.

According to the manufacturer, the Phrozen Mini 8k 3D printer (Phrozen Tech. Co., Ltd., Taiwan) features a very high printing resolution of 8K (7680 × 4320 pixels), which allows for extraordinary sharpness of detail and high precision. In addition, this printer also offers variable printing speed, which allows objects to be produced n a relatively short period of time [27].

The Phrozen Sonic Mighty 8k (Phrozen Tech. Co., Ltd., Taiwan), on the other hand, features the use of high-speed UV LEDs to speed up the resin-curing process. As a result, the Sonic Mighty 8k offers even faster printing times, which is important for those who require high productivity and short production times. In addition, it maintains a high 8K resolution, guaranteeing precise and aesthetically pleasing prints, which is extremely important for dental prostheses and other medical applications [28].

The process of creating the shapes from the selected prosthetic materials was long and consisted of several steps. At first, it was necessary to prepare a suitable CAD model and save it in STL file format. Then, the appropriate printing parameters, such as layer thickness, exposure time, and wavelength, had to be selected to achieve the expected properties of the sample. For the fabricated dogbone shapes, these values were 100 μm layer thickness, 5.8 s exposure time and 405 nm wavelength, respectively. Once the printing parameters were established, the CAD model was transferred to the Phrozen Mini 8k and Phrozen Sonic Mighty 8k 3D printers (Phrozen Tech. Co., Ltd., Taiwan). Since the selected materials, NextDent Denture 3D, NextDent C&B MFH Bleach, and Graphy TC-80DP, are in liquid form, a mixing process was carried out for each of them, using a device from HEXdent to separate sediment from the bottom of the bottle before placing them in the tank. After the mixing time recommended by the manufacturer (for the first mixing, 2.5 h, and subsequent mixing 1.5 h), the resin was poured into the printer tank. The finished printed shapes were placed in an ultrasonic bath, immersing them in ethanol (>90%). They were then dried and placed in an Anycubic Wash & Cure 3 UV chamber for final polymerization, during which the residual monomer was reduced to a minimum and the claimed mechanical properties of the finished product were obtained. This action ensured that the final product met biocompatibility requirements.

### 3.3. Tensile Strength Test

One of the key methods for testing the mechanical properties of materials is the tensile strength test. During this test, various parameters are recorded that describe the characteristics of the material, such as tensile strength, yield strength, relative elongation, Young’s modulus, and Poisson’s ratio. The test procedure involves the progressive stretching of a carefully prepared flat specimen at a set constant speed. To carry out the process, testing machines are used, which are equipped with appropriate jaws for clamping the specimen, a dynamometer for measuring the applied force, and a displacement sensor that records the elongation Δl relative to the initial length of the specimen. By analyzing the recorded values of the force F and the elongation of the specimen Δl, it is possible to obtain the characteristics σ = f(ε) for the test specimens. The shape of the obtained curve depends on the type of material, which makes it possible to infer its mechanical properties.

**Figure 3:**
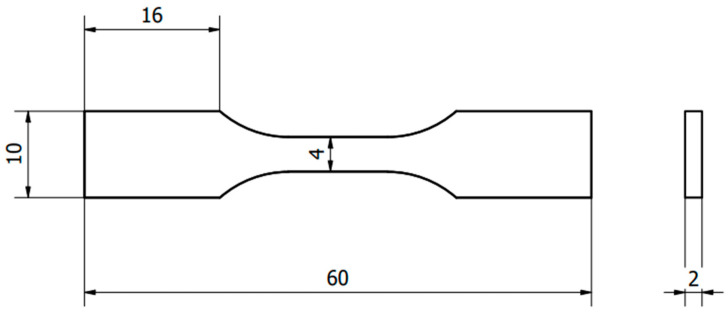
Shape used for tensile strength testing.

In accordance with standard PN-EN ISO 527:1998 on the mechanical properties of synthetic materials during static stretching, the conditions and method of conducting a tensile test of materials are specified [29]. According to this standard, a shape in the form of a “paddle” was adopted, which has certain dimensions: a total length of 140 mm, 90 mm length of the measuring part, 10 mm width of the measuring part, and a thickness of 4 mm with an accuracy of ±0.2 mm. Due to limitations and technological requirements such as the size of the working table of the Phrozen Mini 8k and Phrozen Sonic Mighty 8k printer (Phrozen Tech. Co., Ltd., Taiwan) used, the dimensions of the shaper were scaled. The sample used is shown in Figure 3.

**Figure 4:**
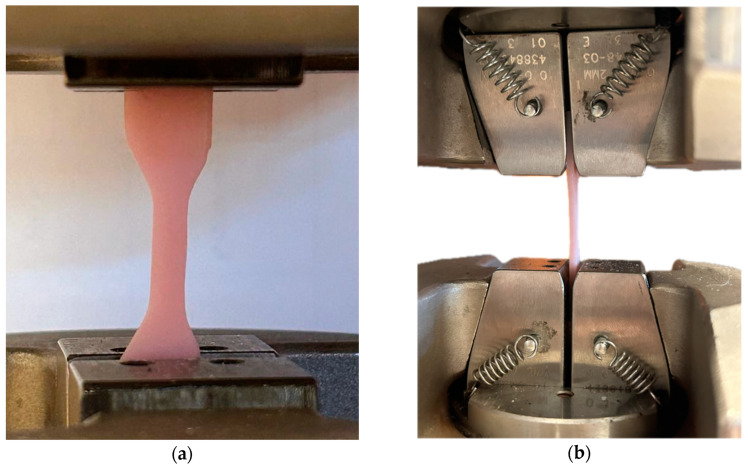
Sample placed in the grips of the MTS testing machine for the static tensile test: (**a**) front view; (**b**) side view.

The MTS testing machine from Bionix was used for the test. Special grips specific to static tensile tests were used to fix the specimen. A material velocity of 5 mm/min was chosen, using the optimal value of displacement length relative to the used dimensions of the shape. Four specimens of each test material (NextDent 3D, NextDent C&B MFH Bleach, and Graphy TC-80DP) were prepared. The specimens were placed symmetrically relative to the grips (Figure 4). For each specimen, only one tensile strength test was conducted, and the test was stopped when the specimen lost integrity.

### 3.4. Preparation of Numerical Model

**Figure 5:**
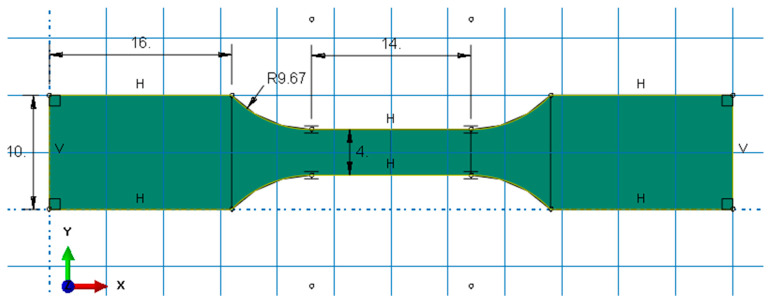
Dimensions of the sample in the Abaqus/Explicit computing environment.

In order to expand the current databases and material libraries used for numerical support in biomedical applications, a number of numerical simulations were also carried out to develop numerical models of materials. For this purpose, first, a numerical model of the paddle sample was developed to validate the static test in a numerical environment. The numerical model of the sample based on the actual dimensions of the sample is shown below (Figure 5).

**Figure 6:**
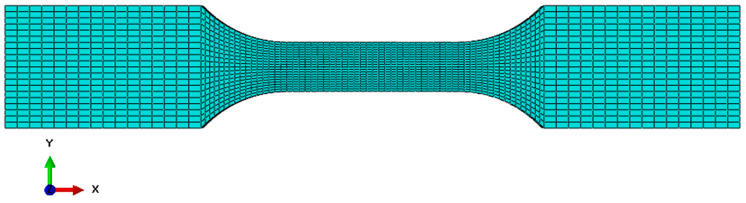
Finite element meshed sample in the Abaqus/Explicit computing environment.

Then, the sample was discretized with hex elements from the Explicit library in the Abaqus/Explicit computing environment. The size of the finite elements was 1 mm in the mounting places and 0.5 mm in the central part of the sample. The tested sample with a finite element mesh applied is shown below (Figure 6).

**Figure 7:**
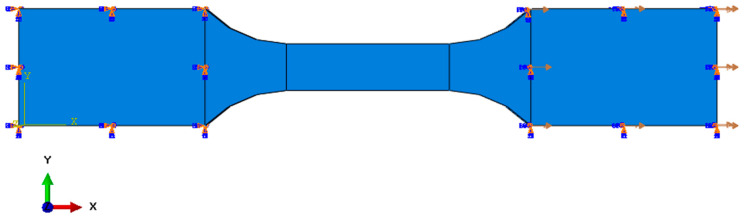
Sample with initial boundary conditions.

Two sample mounting locations were established. For the lower surface of the sample, all translational and rotational degrees of freedom were removed, which corresponds to the stationary mounting location. On the second surface, all rotational degrees of freedom and translational degrees of freedom in the Y and Z axes were removed. The translation in the X axis remained unlocked, which allowed the testing machine to operate at a feed speed of 5 mm/min. The sample with the given initial boundary conditions is presented below (Figure 7).

The Johnson–Cook (1) constitutive model was used to represent the behavior of the material. In the model, at the current stage of research, only the strength strengthening of the material was taken into account, without the terms responsible for the strain rate and temperature. This model is very often used due to the relatively simple determination of material constants and its availability in many computing environments [30,31,32].

(1) $$\sigma_{y}=(A+B{\underline{\varepsilon }}^{p^{n}})(1+Cln{\dot{\varepsilon }}^{*})(1-T^{*m})$$

where *A*—yield strength, *B*—strengthening constant, *C*—strain rate constant, *n*—strengthening exponent, *m*—thermal softening coefficient, ${\underline{\varepsilon }}^{p}$—effective plastic strain, ${\dot{\varepsilon }}^{*}$—effective strain rate (dimensionless), ${\dot{\underline{\varepsilon }}}^{p}$—strain rate, ${\dot{\varepsilon }}_{0}$—reference value for strain rate, T*—homologated temperature (dimensionless), *T_room_*—room temperature, *T_melt_*—melting point, and *T*—current temperature. 

Then, material models were developed using CurveFitter 2024 software. Thanks to the software, the parameters of the J–C model were adjusted based on the data from the experimental test (static tensile test). The determined material parameters used to describe the behavior of individual materials tested experimentally are presented below (Table 5).

Limit strain with SPH particle hydrodynamics was adopted as the failure model. The strain value at the breaking of the material corresponds to the average strain value at which the sample was torn during experimental tests. These values are summarized in the table below (Table 6).

Then, the material data were applied to the computational environment and simulations were performed for each case to validate the developed material models.

## 4. Tensile Strength Test Results

**Figure 8:**
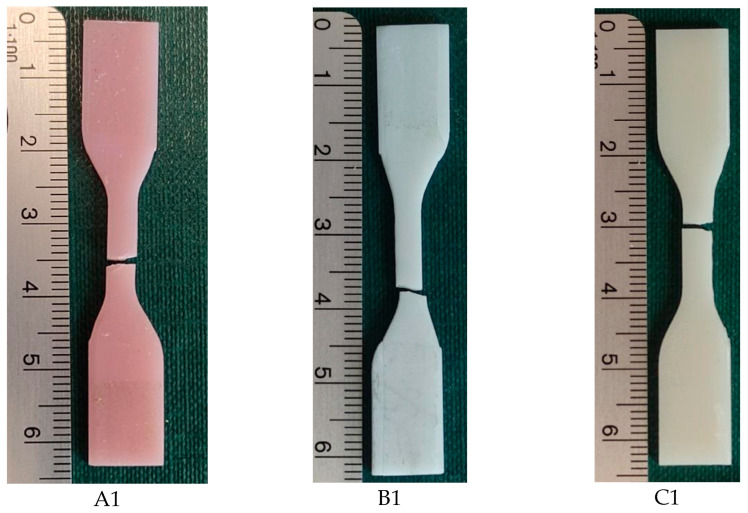
Samples after static tensile test: (**A1**)—NextDent Denture 3D, (**B1**)—NextDent C&B MFH Bleach, (**C1**)—Graphy TC-80DP.

**Figure 9:**
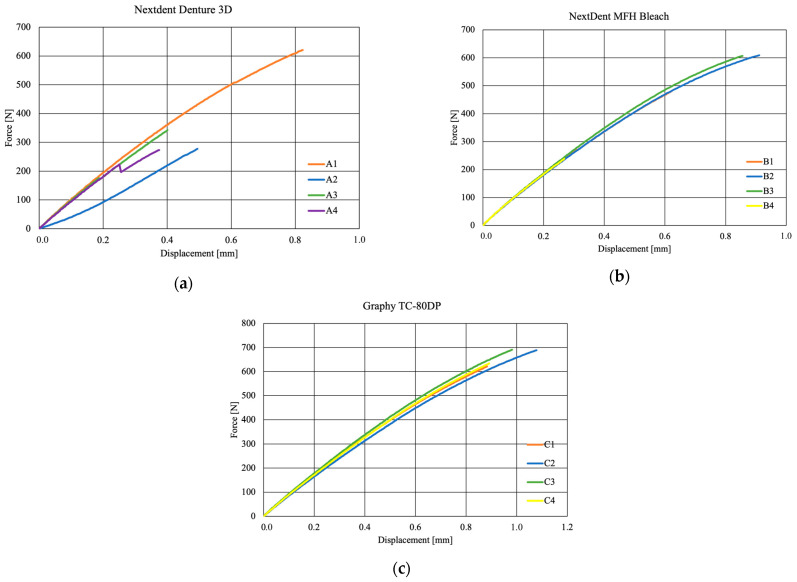
Material characteristics: (**a**) NextDent Denture 3D, (**b**) NextDent C&B MFH Bleach, (**c**) Graphy TC-80DP.

Figure 8 shows the locations of the loss of shape integrity for 1 sample per material. For the three selected photopolymer materials for use in incremental technologies, the obtained characteristics σ = f(ε) from the static tensile test were compared (Figure 9). The series for the tests of the NextDent manufacturer’s materials contained 4 samples each, and for the Graphy brand, 5 tensile tests were carried out. The obtained results were compared with each other, and on the basis of the values shown in the graphs, appropriate conclusions were drawn about the properties of the tested materials.

The graphs above show a model of rheology characterized by a linear part up to the 250 N range and a plastic part up to values above 600 N. The visible displacement of 0.8 mm for each specimen indicates equal strength, while force values of 600 N and 700 N were obtained for NextDent and Graphy materials, respectively. The characteristic faults shown in Figure 9a can be considered material defects accrued by the material during the manufacturing process, i.e., possible sedimentation of the ceramic additive or manufacturing inaccuracy, which resulted in additional stresses during testing.

## 5. Material Analysis

### 5.1. Microstructure Analysis

Material structure research was performed by SEM (scanning electron microscopy) using a Hitachi TM-3000 (Hitachi Ltd, Chiyoda, Tokio, Japan) scanning microscope coupled to an EDS (energy-dispersive spectroscopy) analyzer. The studies were conducted using an SE (secondary electron) detector at an accelerating voltage of 15 kV. The tests were carried out on the surface of samples subjected to preparation consisting of preliminary grinding on water-based sandpaper and polishing on polishing cloths. Finally, the surfaces of the samples were subjected to a preparation consisting of coating the surface with graphite using a vacuum sputtering system with a Q150T turbomolecular pump.

**Figure 10:**
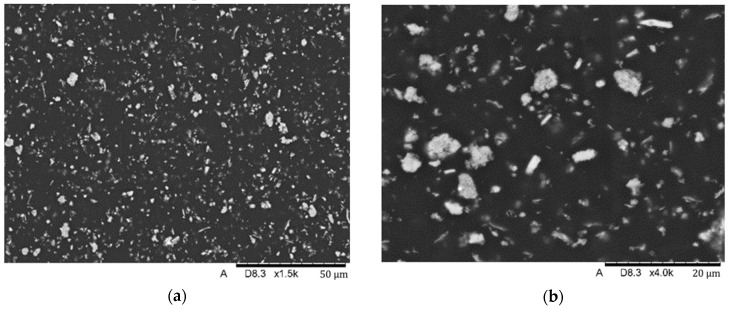
SEM images of the microstructure of Nextdent 3D material. Magnification: (**a**) 1500×; (**b**) 4000×.

**Figure 11:**
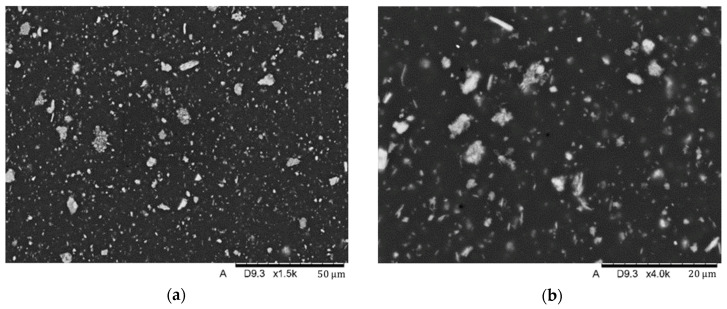
SEM images of the microstructure of NexDent MFH Bleach material. Magnification: (**a**) 1500×; (**b**) 4000×.

SEM analysis showed the presence of fillers in the matrix of the samples Nextdent 3D (A) and NextDent C&B MFH Bleach (B), which was marked by a light gray color against the dark matrix. No filler elements were found in the Graphy TC-80DP (Graphy Inc., Seoul, Republic of Korea) (C) samples. A general view of the materials’ structure is shown in Figure 10 and Figure 11.

**Figure 12:**
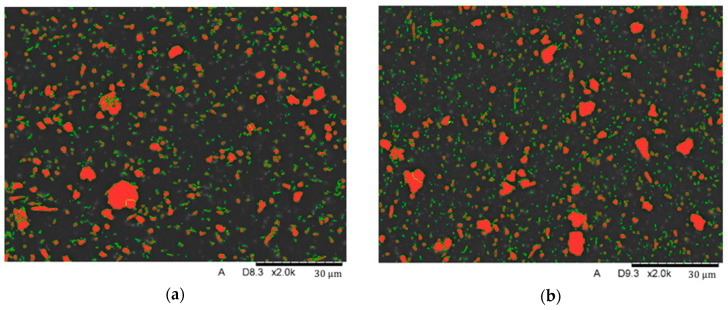
Image analysis: binary images of the surface using a magnification of 2000×: (**a**) Nextdent 3D; (**b**) NextDent C&B MFH Bleach.

Tests were carried out on the surface of the grinds using image analysis methods to determine the proportion of filler elements in the structure. Image analysis was carried out using the Nikon NIS Elements system. Figure 12 shows examples of the binary images used in the study.

The tests showed that both Nextdent 3D (A) and NextDent C&B MFH Bleach (B) materials have filler elements in the form of fine-dispersed particles with sizes ranging from 0.1 mm to 5 mm. Larger elements reaching about 12 mm in size are also noticeable in the matrix. They are most often an agglomerated group of several to even dozens of particles of smaller sizes. The study determined that Nextdent 3D contains an average of 9.2% and NextDent C&B MFH Bleach 10.3% of filling elements, with Nextdent 3D containing an average of 97.8% of particles up to 5 mm and NexDent MFH Bleach 96.2%. The remainder is made up of particles with larger sizes.

**Figure 13:**
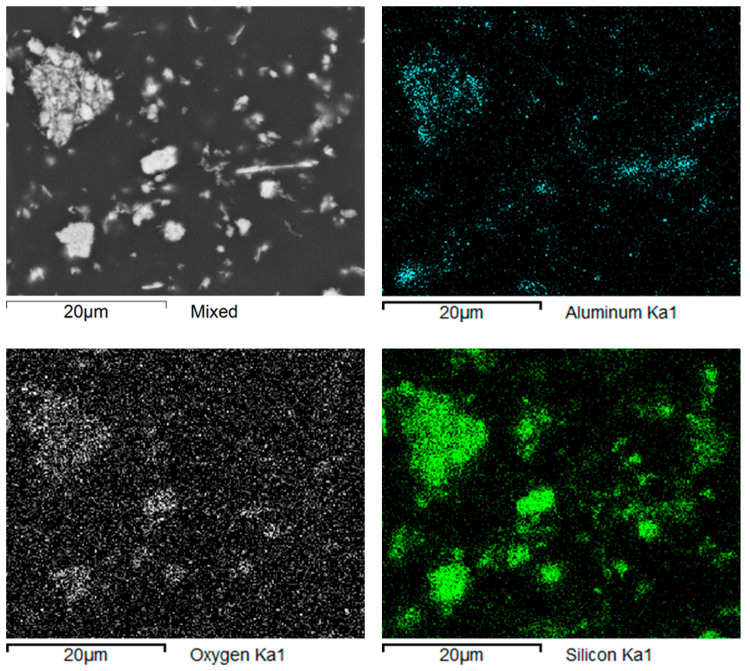
Nextdent 3D: maps of elements’ distribution in the composite’s structure.

**Figure 14:**
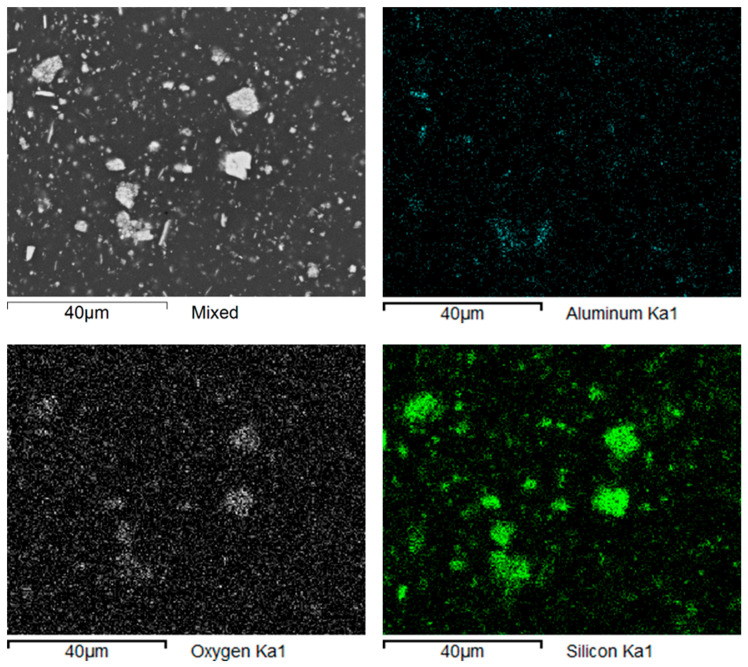
NexDent MFH Bleach: maps of elements’ distribution in the composite’s structure.

The material structure study was followed up with a mapping analysis showing the distribution of dominant elements on the ground surfaces of the studied samples. In both the Nextdent 3D and NextDent C&B MFH Bleach materials, the reinforcing elements were found to be mostly compounds of elements such as silicon Si, aluminum Al and oxygen O. Examples of the analysis results are shown in Figure 13 and Figure 14.

**Figure 15:**
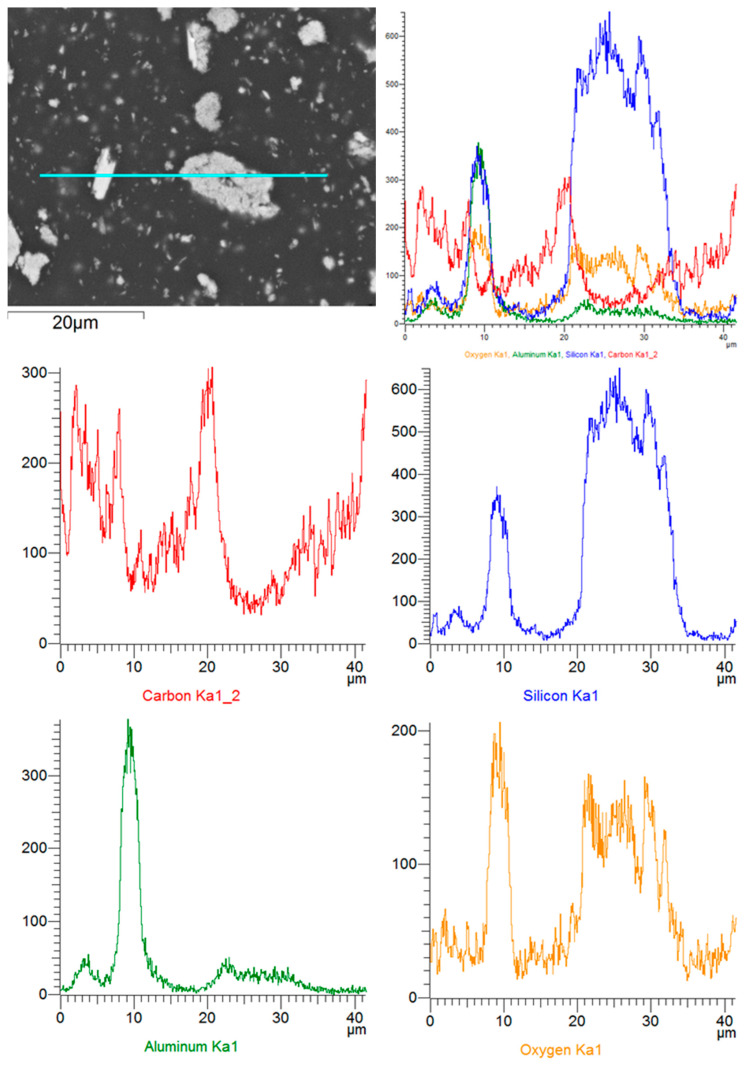
NexDent MFH Bleach: linear analyses of elements’ distribution in the composite’s structure.

During further research identifying the elements that make up the filler elements used in the materials, linear analyses were performed along selected scan lines. The results of these studies are shown in Figure 15.

**Figure 16:**
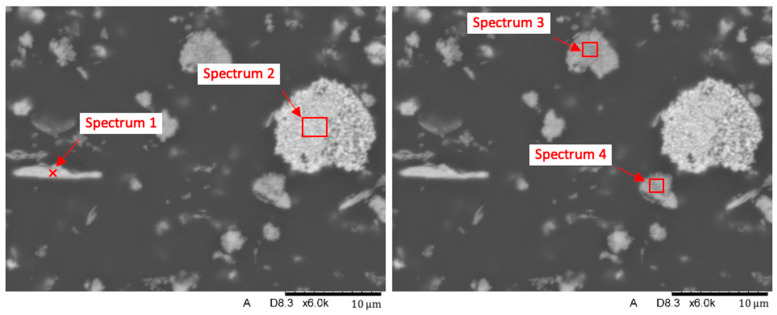
Nextdent 3D: linear analyses of elements’ distribution in the composite’s structure.

**Figure 17:**
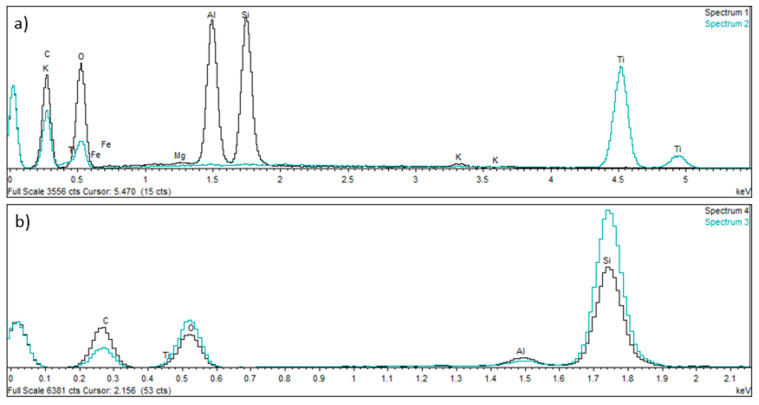
Spectral analysis of selected elements in the Nextdent 3D material structure: (**a**) spectrum 1 and 2; (**b**) spectrum 3 and 4.

**Figure 18:**
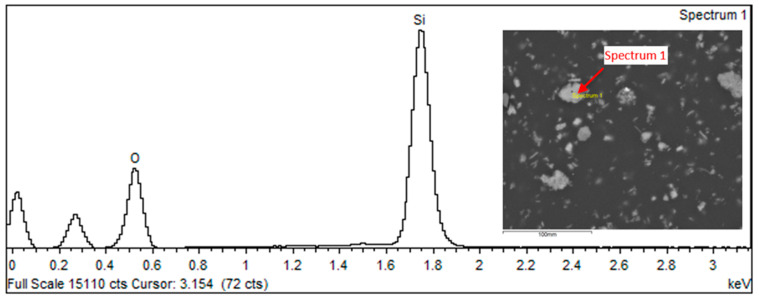
Spectral analysis of selected elements in the Nextdent 3D material structure: spectrum 1.

**Figure 19:**
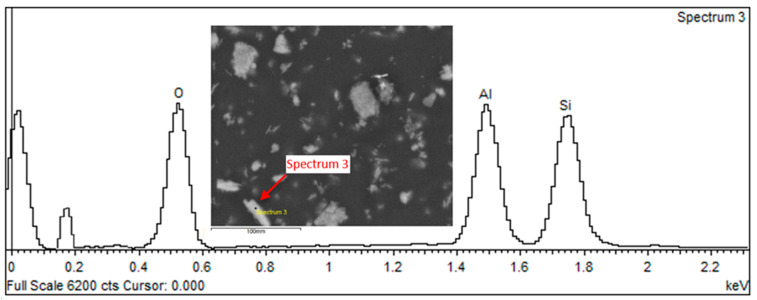
Spectral analysis of selected elements in the Nextdent 3D material structure: spectrum 3.

The results of the chemical EDS spot and area analyses (Figure 16, Figure 17, Figure 18 and Figure 19 and Table 7, Table 8 and Table 9) on the surface of the filler elements on the grinds of the samples prepared for testing found that the main filler was made up of SiO_2_ silica particles. They constitute about 55–60% of the contribution. The remaining fraction was identified as aluminum silicates (Al_2_O_3_· SiO_2_). It is important to note that SiO_2_ elements occur as finely dispersed particles and in the form of agglomerates, while Al_2_O_3_ SiO_2_ elements occur in the form of flakes. The results of the tests with the determination of the weight composition of the areas marked in Figure 16 are shown in Figure 17 and Table 7.

The conducted analysis confirmed the trace presence of Ti (Spectrum 2) in the chemical composition of titanium fillers, which occurs in the oxygen O-bound form (Ti-O). Closer identification pointed to the TiO_2_ compound.

Figure 18 and Figure 19 as well as Table 8 and Table 9 show the results of the identification analysis including the composition by weight and the atomic composition of the particles constituting the main fractions of the composite filling.

### 5.2. SEM Analysis of the Fracture Surface

**Figure 20:**
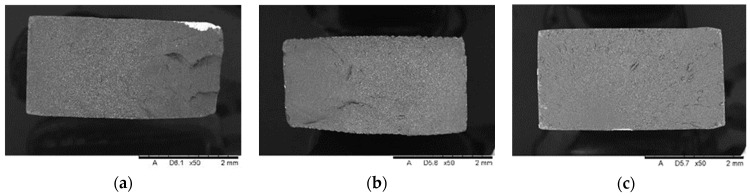
General view of the face of the reference breakthroughs of the specimens after static tensile testing: (**a**) Nextdent 3D (A1); (**b**) NexDent MFH Bleach (B1); (**c**) Graphy TC-80DP (C1).

Figure 20 shows a general view of sample reference breakthroughs obtained after tensile testing.

**Figure 21:**
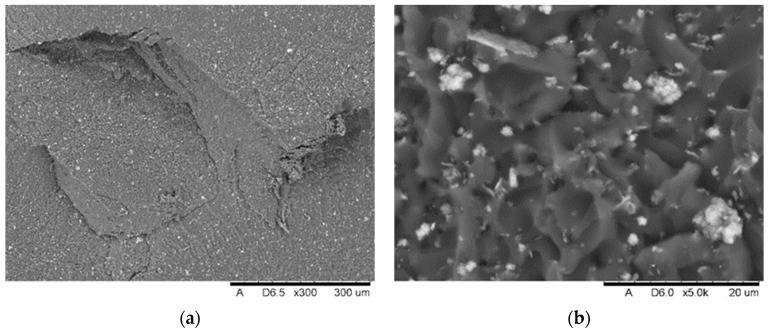
View of the fracture front surface of the Nextdent 3D specimen (A1): (**a**) visible in the surface topography is a system of high faults associated with the passage of the fracture front through an area of local micro-deformation. (**b**) View of filler particles on the fracture surface.

**Figure 22:**
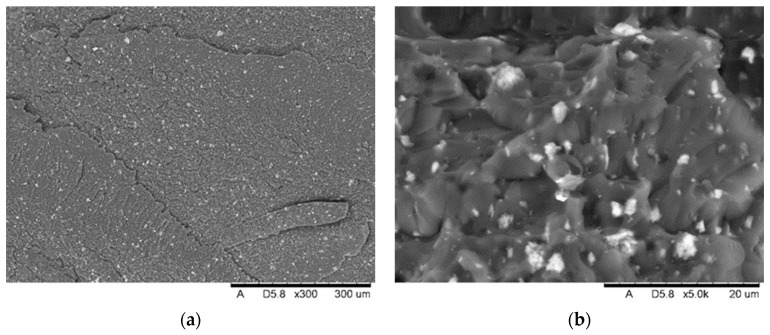
View of the fracture front surface of the NexDent MFH Bleach (B1): (**a**) visible in the to-pography of the surface is a system of low faults associated with the passage of the fracture front through an area of local micro-deformation, (**b**) view of filler particles on the surface of the breakthrough.

**Figure 23:**
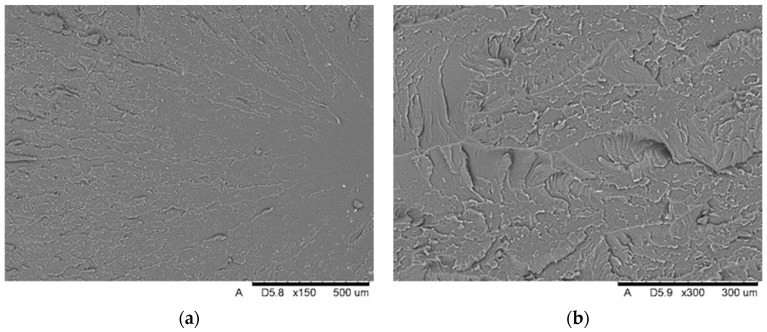
View of the fracture surface of the Graphy TC-80DP (C1) specimen: (**a**) a system of low faults around the fracture focus visible in the surface topography; (**b**) a view of material pullouts on the fracture surface.

The results of SEM observations revealing the topography of the breakthroughs are shown in Figure 21, Figure 22 and Figure 23. The analysis showed that both the Nextdent 3D and NextDent C&B MFH Bleach specimens (Figure 21 and Figure 22) had visible systems of high faults, usually located near one of the shorter edges of the specimen surface in the surface topography. Low faults are visible in the rest of the breakthroughs. The surfaces show numerous material pullouts, with the presence of micro-cracks causing fragmentation. On the surface of the cracks, observations confirm the presence of filler particles strongly bonded to the matrix material.

On the breakthroughs of the specimens of the Graphy TC-80DP material (Figure 23), we observed strongly outlined outbreaks with a smooth surface and radially spreading lines outlining the direction of crack propagation. The crack lines show shallow micro-fractures associated with the passing of the crack front through an area of local micro-deformation. Cracking along the faults is accompanied by moderately ductile material deformation.

## 6. Numerical Analysis and Results

**Figure 24:**
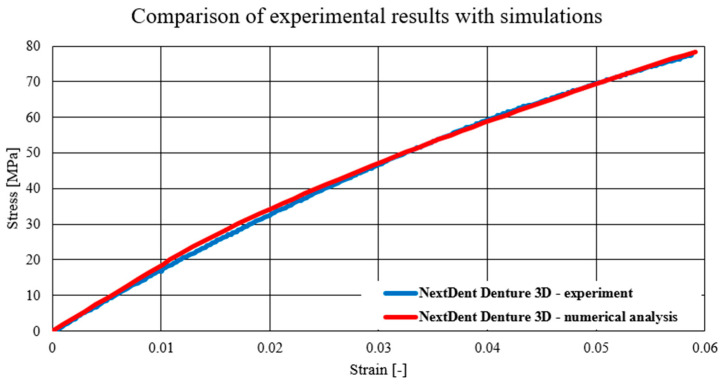
Comparison of experimental results with simulations—NextDent Denture 3D.

**Figure 25:**
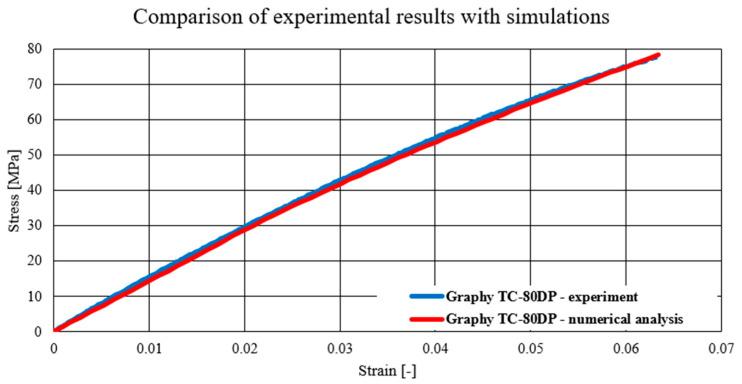
Comparison of experimental results with simulations—Graphy TC-80DP.

**Figure 26:**
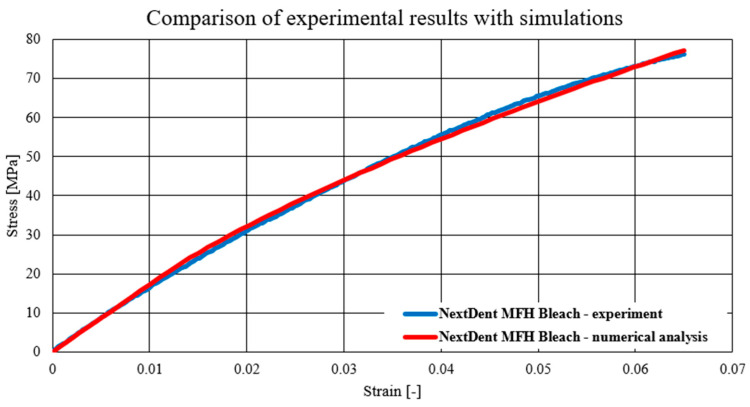
Comparison of experimental results with simulations—NextDent MFH Bleach.

The charts below present a comparison of experimental data with data obtained as a result of numerical simulations. The first material model verified was the NextDent Denture material model, which is presented in the chart below (Figure 24). Subsequently, verification was also carried out for the materials Graphy TC-80DP (Figure 25) and NextDent MFH Bleach (Figure 26).

**Figure 27:**
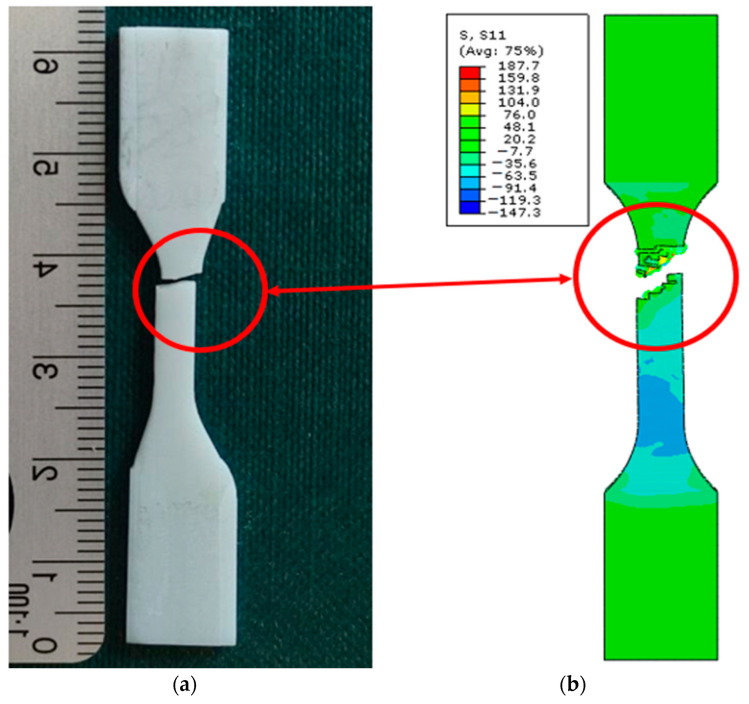
Fracture comparison of samples: (**a**) real sample; (**b**) sample in a computational environment.

As a result of numerical simulations, a high correlation with our experimental results was obtained, with over 98% coverage of the values of all graphs. An analysis of the fracture mechanics of the samples in a numerical environment was also performed in comparison to the experimental results. In this case, a satisfactory correlation was also obtained. The samples in the numerical environment cracked in places similar to the real samples. Below (Figure 27) is an example of a sample crack obtained for the Graphy TC-80DP material.

**Figure 28:**
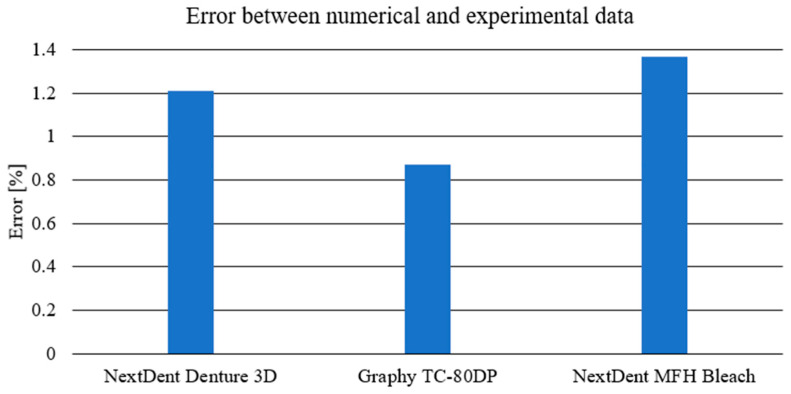
Comparison of obtained error between the experimental and numerical data for chosen materials.

The performed numerical tests allowed for chart coverage in each case to be obtained at a 98% level. To determine the discrepancies between the experimentally obtained curves and the material characteristics determined on the basis of numerical analyses, a method was used to identify the intersection points of the compared curves. The intersection points mark the boundaries of the areas for which the area under the curves is calculated by integration. The difference in the values of these areas for each region determines the local discrepancy between the analyzed curves (Figure 28).

This constitutes an important contribution for future research, including those enabling the extension of the numerical model with terms responsible for the influence of strain rate and temperature. Additionally, verified material models can be successfully used to simulate dental elements (e.g., prosthesis on implants).

## 7. Discussion

Mechanical and material analysis of selected biomedical materials used in implantology is a key element in assessing their suitability and properties. The research carried out using SEM electron microscopy revealed important structural features and chemical composition of the materials, which allowed for a deeper understanding of their physical properties that will be useful for further steps towards better implant design.

The results of the structure analysis of Nextdent 3D (A) and NextDent C&B MFH Bleach (B) materials indicated the presence of filler elements in the form of finely dispersed particles with sizes ranging from 0.1 mm to 5 mm. It was noted that the proportion of these elements and their sizes varied among the materials tested. Detailed chemical analyses by spot and area EDS analysis identified the main filler components, i.e SiO_2_ silica particles and aluminum silicates (Al_2_O_3_·SiO_2_). Significantly, these components are present in different forms, which can affect the stability and strength of the materials. It is worth noting that Graphy TC-80DP (C) samples did not show the presence of filler elements.

Analysis of the surface topography of the specimens revealed distinctive features such as the presence of faults and microcracks, which may be important for assessing the resistance to mechanical forces and the stability of the materials under clinical conditions. Systems of high faults, usually concentrated around one of the shorter edges of the specimens, were observed on the surfaces of the Nextdent 3D and NextDent C&B MFH Bleach materials. In addition, numerous material pullouts were observed on the surfaces, which were often associated with microcracks, leading to material fragmentation. The tests also confirmed the presence of filler particles, which were closely associated with the matrix material, on the surfaces of the cracks. The fractures of the samples made of Graphy TC-80DP material presented other characteristic features. Strongly outlined foci with a smooth surface were observed, with radially spreading lines that marked the direction of crack propagation. Shallow micro-fractures could be seen along the crack lines, which were associated with the crack front passing through an area of local micro-deformation. In addition, cracking along the faults was accompanied by moderately ductile deformation of the material. This knowledge may influence the clinician’s decision to choose a specific resin for planned reconstructions. As for the immediate loading of implants in all-on-X cases, it is important to produce a rigid and more resilient construction (e.g., with Graphy resin) in order to provide proper stabilization. Otherwise, implants will not osseointegrate and the patient will lose them. On the other hand, in certain cases, the clinician only needs to maintain proper occlusion and function of mastication while waiting for tissues to heal before implementing the main treatment plan. In these cases, a less rigid temporary prosthesis (made with NextDent resins) is more than enough and may be more economical for the patient. 

The high correlation (at 98%) of the results of numerical simulations, wherein the Johnson–Cook model of elastic–plastic materials was used, with experimental results is an important confirmation of the effectiveness of the research methods and material models used. This could be crucial for further research on optimizing the properties of biomedical materials and their application in clinical practice, including for simulation of dental components. The numerical study also confirmed the correlation between the fracture of the real sample and that of the computational environment. This means that the use of the deformational failure criterion with SPH particle conversion is correct in the context of mapping the behavior of materials used in biomedical practice. In many existing studies [33,34,35,36,37], the authors have focused on conducting numerical analysis in static ranges without taking into account fracture mechanics. This has allowed for the prediction of stresses occurring in simplified conditions, but cannot predict, for example, the development of cracks. The use of dynamic models, which are part of this work, will allow the in vivo behavior of printed elements to be simulated, e.g., during chewing, which will allow us not only to predict occurring stresses and deformations but also to predict the possible propagation of cracks under existing conditions.

In the discussion of how prosthetic replacements behave under different conditions, an important aspect to consider is the influence of the materials they are made of on their mechanical properties and load response. While dentures made from different biomaterials may exhibit different load responses, standardized tests such as tensile strength testing are needed to objectively assess their strength and behavior under clinical conditions. The final shape of the denture or bridge and individual patient-dependent factors have a significant impact on the lifespan of the device, but in the context of the study conducted, the main focus was on material aspects in order to understand the mechanical properties of the selected materials: NextDent Denture 3D, NextDent C&B MFH Bleach, and Graphy TC-80DP.

The selection of specific test methods is crucial; this allows us to objectively evaluate the materials and their mechanical properties. This allows us to better understand which materials are most suitable for use in different clinical situations, which contributes to the standardization of procedures and improves the quality and durability of restorations.

## 8. Conclusions

- The proper selection of material models in the computer environment had a key impact on the final results obtained. In this case, the use of the Johnson–Cook model allowed a reliable representation of the behavior of resins used in dentistry, showing a 98% correlation between experimental results and numerical simulations. In addition, the combination of the SPH method with the standard FEM in the limit deformation task allowed for a very good match in fracture mechanics in terms of discontinuities in the structure of the geometric systems considered for the three resins.
- Significant differences were observed between the Young’s modulus values for the tested materials (Table 10). The values obtained in the tensile strength tests are different from the values reported by the producers in the data sheets. In fact, the tested materials have significantly lower Young’s modulus values than those reported by the producers.
- The mechanical and material studies conducted provide important information on the structure, chemical composition, and mechanical properties of the selected NextDent Denture 3D, NextDent C&B MFH Bleach, and Graphy TC-80DP biomedical materials used in implantology. The high correlation between experimental results and numerical simulations provides a solid basis for further research into the optimization of these materials and their clinical application.
- The presence of filler elements such as SiO_2_ silica particles and aluminum silicates (Al_2_O_3_ SiO_2_) in the NextDent Denture 3D+ and NextDent C&B MFH Bleach materials significantly affected the mechanical properties of the biomedical materials tested. These micronutrients strengthened the material’s structures by forming a network structure around them, leading to increased stress and fracture resistance. In addition, differences in the proportion and size of these elements affected the density, hardness, and Young’s modulus of the material.
- In Graphy TC-80DP (C) samples, there were no filler elements found to directly affect the strength, so the best mechanical strength value was due to the homogenization of the material structure.
- The ceramic additive applied to the resin base significantly improved mechanical properties such as hardness and abrasive wear and increased the strength limit, which directly improved the mechanical properties of the finished product. The applied material can be considered a polymer–ceramic composite, so it will be possible to optimize its percentage to improve its mechanical and technological properties during 3D printing by reducing the sedimentation phenomenon characteristic of ceramic particles in resins.
- Analysis of the surface topography of the samples revealed characteristic features such as faults and micro-cracks, which may be important for assessing the resistance of the materials to mechanical forces and their durability in long-term use. The cracks showed a concentration of stresses on the ceramic particles, and the crack line followed their boundaries.
- At a later stage in this study, tests should be carried out on completed products made from the selected materials in order to verify the results obtained under near-real conditions.

## Figures and Tables

**Table 1:** Mechanical properties of NextDent Denture 3D (NextDent B.V., Soesterberg, The Nederlands) material.

Material	E [MPa]	ρ [g/cm^3^]	σ_g_ [MPa]
NextDent Denture 3D	2383.0	1.26	84.0

**Table 2:** Mechanical properties of NextDent C&B MFH Bleach material.

Material	E [MPa]	ρ [g/cm^3^]	σ_g_ [MPa]
NextDent C&B MFH Bleach	-	1.2	107.0

**Table 3:** Mechanical properties of Graphy TC-80DP material.

Material	E [MPa]	ρ [g/cm^3^]	σ_g_ [MPa]
Graphy TC-80DP	4500.0	1.07	220.0

**Table 4:** Mechanical properties of acrylic resin.

Material	E [MPa]	ρ [g/cm^3^]	σ_g_ [MPa]
Acrylic	1603.0	1.1	69.8

**Table 5:** Obtained material parameters based on experimental research.

Material	*E* [MPa]	*v* [-]	*ρ* [kg/m^3^]	*A* [MPa]	*B* [MPa]	*n* [-]
NextDent Denture 3D	1847	0.4	1260	20.11	632.51	0.582
Graphy TC-80DP	1437	0.4	1070	27.01	694.24	0.551
NextDent MFH Bleach	1732	0.4	1200	19.59	538.54	0.575

**Table 6:** Adopted failure strain values.

Material	Failure Strain [-]
NextDent Denture 3D	0.063
Graphy TC-80DP	0.068
NextDent MFH Bleach	0.070

**Table 7:** Chemical composition [% wt.] of spectra from selected surface areas.

Name	C *	O	Mg	Al	Si	K	Ti	Fe
Spectrum 1	41.890	37.748	0.114	8.675	10.618	0.418		0.537
Spectrum 2	27.743	33.697					38.560	
Spectrum 3	33.899	40.395		0.626	25.079			
Spectrum 4	48.033	34.607		1.178	16.026		0.156	

Note: * values from the determination of carbon C follow from the presence of this element in the matrix of the material.

**Table 8:** Chemical composition of spectra from selected surface area of spectrum 1.

Element	Weight%	Weight% σ	Atomic%
Oxygen	23.411	0.176	34.921
Silicon	76.589	0.176	65.079

**Table 9:** Chemical composition of spectra from selected surface area of spectrum 3.

Element	Weight%	Weight% σ	Atomic%
Oxygen	34.632	0.407	46.234
Aluminum	33.325	0.362	29.121
Silicon	32.043	0.310	24.645

**Table 10:** Comparison of Young’s modulus values of the experimental method and producers’ parameters.

Material	E_experimental_ [MPa]	E_catalog_ [MPa]
NextDent Denture 3D	1847	2383
Graphy TC-80DP	1437	4500
NextDent MFH Bleach	1732	-

## Data Availability

The data supporting reported results can be obtained upon request from author D.P.
